# An Update on Translating Stem Cell Therapy for Stroke from Bench to Bedside

**DOI:** 10.3390/jcm2040220

**Published:** 2013-11-01

**Authors:** Travis Dailey, Christopher Metcalf, Yusef I. Mosley, Robert Sullivan, Kazutaka Shinozuka, Naoki Tajiri, Mibel Pabon, Sandra Acosta, Yuji Kaneko, Harry van Loveren, Cesar V. Borlongan

**Affiliations:** Center of Excellence for Aging & Brain Repair, Department of Neurosurgery and Brain Repair, University of South Florida Morsani College of Medicine, 12901 Bruce B. Downs Blvd., Tampa, FL 33612, USA; E-Mails: tdailey@health.usf.edu (T.D.); cmetcalf@health.usf.edu (C.M.); ymosley@health.usf.edu (Y.I.M.); rsulliv1@health.usf.edu (R.S.); kshinozu@health.usf.edu (K.S.); ntajiri@health.usf.edu (N.T.); mpabon@health.usf.edu (M.P.); sacosta@health.usf.edu (S.A.); ykaneko@health.usf.edu (Y.K.); hvanlove@health.usf.edu (H.L.)

**Keywords:** stem cells, stroke, transplantation, translational biomedical research

## Abstract

With a constellation of stem cell sources available, researchers hope to utilize their potential for cellular repair as a therapeutic target for disease. However, many lab-to-clinic translational considerations must be given in determining their efficacy, variables such as the host response, effects on native tissue, and potential for generating tumors. This review will discuss the current knowledge of stem cell research in neurological disease, mainly stroke, with a focus on the benefits, limitations, and clinical potential.

## 1. Translational Gating Items of Stem Cell Therapy

With the increasing diversity of stem cell sources emerging for donor cells in transplantation therapy, many laboratory-to-clinic translational factors must first be considered, dynamics such as the source of the cells, ease of extraction, immunogenicity, capacity for proliferation, and cell yield. These concerns may serve as potential limitations respective to the donor cell origin being considered, proving a particular source to be a more suitable therapy for a specific disease.

Harvesting of stem cells may be divided into two domains, allogenic *vs*. autologous sources (though xenogeneic cells have been previously tested). Autologous stem cells are acquired from the host in which the cells are intended for use, while allogenic cells are procured from an unrelated donor prior to transplantation. As one may expect, the use of allogenic stem cells may predispose an individual to various immunologic complications upon treatment, giving rise to the significant limitation of graft rejection with this method of treatment. Yet, autologous treatments may be limited by their ability for propagation and cell yield. 

The immunological barriers, such as graft *vs*. host or required immunosuppression of the host, are of constant consideration in the therapeutic benefits and limitations of stem cell transplantation. Prior research of immunocompromised stroke animals demonstrated inhibition neurogenesis in the cortex endogenously via a CD4^+^ T cell, but not CD25^+^ T cell mechanism, supporting the influence of immunodeficiency in reducing stem cell apoptosis [1]. In another study, upon exposure to cyclosporine A, an immunosuppressant, there was enhanced recovery of cortical injury following stroke secondary to endogenous stem cell activity and migration [2]. These papers support the hypothesis that inflammation following a cerebral event may not only damage tissue, but also further disrupt the endogenous neurogenic pathways of repair involving the migration of stem cells from the lateral ventricle regions of the brain, a topic further discussed in the neural stem cell section. This contrasts the proposed mechanism of action for neurotrophic modulators that are believed to furnish a microenvironment conducive to repair, but do not yield new neurons [2].

Because of the potential immunological host response to the transplanted cells, much research has been conducted investigating specific cell line’s prospective immunogenicity. Surmounting evidence suggests that the more naive the cell lineage, the less likely the incidence of immunological reaction following transplantation. For instance, umbilical cord blood, due to its immunological immaturity, is less likely to invoke an immunological response and therefore less likely to require immunosuppression. More so, human leukocyte antigen (HLA) matching may be less stringent in umbilical cord blood transplants compared to bone marrow derived transplants and even whole tissue grafts, leading to higher cell viability [3]. Contrasting immunogenicity, some cell lineages may be more immunosuppressive than bone marrow derived stem cells. HLA-G, a contributing factor of immunosuppression [4], is of higher expression in chorionic plate-derived mesenchymal stem cells compared to bone marrow-derived stem cells and adipocyte tissue-derived mesenchymal stem cells [5], suggesting its usefulness as a prognostic indicator of transplant viability in the presence of host immunity [6]. Additionally, placenta-derived mesenchymal stem cells demonstrate less immunomodulation than bone marrow-derived mesenchymal stem cells, suggesting regenerative transplantation potential to be less efficacious [7]. 

## 2. Tailoring Stem Cells for Therapeutic Applications in Stroke

Stroke is a major unmet clinical need with only one current Food and Drug Administration (FDA)-approved drug, the tissue plasminogen activator, efficacy limited to 4.5 h after stroke onset. Accordingly, the challenges of proliferation capacity and cell yield are evident with regards to the optimum delivery time of stem cell therapy. With the current clinical trials of cell therapy for acute stroke mostly targeting a window of 48 h, the potential limitation in generating a sufficient number of autologous cells from freshly harvested tissue for therapy in such a short period is apparent [8]. In view of this limitation, allogeneic transplantation is indicated when contemplating with acute stroke therapy. Alternatively, the extended time required for cell amplification with autologous stem cell transplantation renders it more appropriate for chronic stroke therapy. Nonetheless, regardless of autologous or allogeneic stem cell sources, cell harvesting imparts additional technical challenges. For example, acquiring neural stem cells may require invasive procedures that may be disadvantageous despite the therapeutic potential of the cells.

The following sections aim to outline the different tissue sources available for harvesting stem cells, along with their respective benefits, limitations, and prospective use in clinical application as they pertain mainly to neurological diseases, most notably stroke therapy. A review of current stem cells being investigated in neurorestoration has recently been published [9]. We only briefly discuss embryonic and extraembryonic stem cells and focus this paper on our long-standing research interest in adult stem cells.

## 3. Searching for Safe and Effective Stem Cell Therapy

Embryonic stem cells are pluripotent cells derived from the inner cell mass of the blastocyst that arguably serve as the foundation by which the properties of “stemness” are measured in other cell lines. With the potential to differentiate into all three germ layers, transplantation of embryonic stem cells (ESCs) into animal stroke models has demonstrated repair in both vascular [10] and neuronal damage [11], improved functional recovery of deficits [12,13,14,15,16] and provision of neurotrophic, angiogenic, and anti-apoptotic effects [13,14,15,16,17]. These benefits may extend well into potential translational therapy following a cerebral event, giving the cell line a plentiful array of therapeutic actions in terms of modulating a number of diseases including stroke. The distribution of these cells has been demonstrated with imaging techniques in both the brain and the periphery following transplantation in animal stroke models [18,19]. Although ESCs possess the potential for vast differentiation, there are two predominant concerns limiting their use. The ethics of harvesting embryonic stem cells is widely debated. However, recent advancements, discussed later in this review, may further develop and refine methods for producing these cells through retrograde manipulation of mature cell lines, alleviating some of the tension surrounding their use. Additionally, their naive lineage aligns with the stem cell tenet of the more naive the cell, the greater the potential for tumorigenicity, a topic that will be expanded upon in subsequent sections. Stem cells have the potential to form tumors after transplantation. This tumorigenicity is mostly associated with embryonic stem cells and pluripotent stem (iPS) cells [20]. Shortly after transplantation, a dysregulated differentiation of ESCs was found to cause the formation of teratomas containing all types of somatic tissues of the early embryo [21], due likely to the presence of oncogenes and trisomies which are known to have roles in cancer cell formation [22]. In view of this stem cell tumorigenicity, strategies have been explored including predifferentiation of cells to remove stemness or genetic modification to activate anti-oncogenic genes such as Nurr1 to abrogate the neoplastic state or render the cells post-mitotic [23,24]. While this tumorigenicity has been closely associated with ESCs and iPS cells, recent evidence suggests that safety precautions should also be taken with adult stem cells due to possible ectopic tissue formation seen in grafted mesenchymal stem cells (MSCs) in the Central Nervous System (CNS) [25]. These studies highlight a major hurdle in stem cell therapy, and emphasize the importance of closely monitoring stemness and tumorigenicity as we translate cell therapy to the clinic [22,24,26].

With the concerns surrounding the use of embryonic tissue to harvest stem cells, researchers have looked to sources external to the embryo to harvest stem cells. Wharton’s jelly (within the umbilical cord), amnion, placenta, and umbilical cord are all rich stem cell sources [27]. As seen with the neural stem cells and mesenchymal stromal cells, extraembryonic stem cells also relate to different germinal layers. The amniotic epithelium is of ectoderm origin while the amnion-derived mesenchymal stromal cells are derived from the mesoderm [28]. Yet, the amnion-derived mesenchymal stromal cells exhibit less endothelial propensity conferring cell specificity [29]. Transplantation of placenta-derived mesenchymal stromal cells in animal models of stroke are believed to supply a microenvironment favorable for endogenous neural repair and replace damaged tissue [30,31,32]. Mesenchymal stromal cells derived from umbilical cord lining develop an immunosuppressive effect while demonstrating functional recovery, increased vascular density, increased expression of vascular endothelial growth factor, and basic fibroblast growth factor in rat stroke models [33,34].

**Figure 1:**
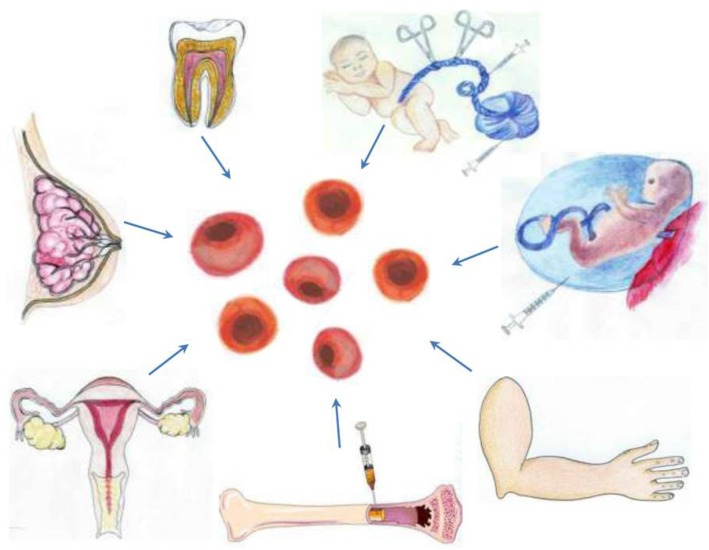
Adult stem sources include umbilical cord blood, placenta, amniotic fluid, bone marrow, menstrual blood, breast milk, dental pup, and skin fibroblasts. Most of these cells have been shown to exert neuroprotective effects in stroke animal models and a few have reached clinical trials in stroke patients.

Adult stem cells often exist in combination with non-stem cells committed to distinct lineage, creating a heterogeneous environment. Because of this, one challenge in the use of adult-derived stem cells is the purification for the isolation for the particular stem cell of interest. In the following sections we will discuss the variety of adult-derived stem cells (Figure 1), their tissue sources, benefits, limitations, and clinical relevance.

### 3.1. A Stem Cell Source with a Long History of Transplant Use: Bone Marrow-Derived Stem Cells

A divergent population stem and blast cells constitute the bone marrow. Thus, these cells may be utilized as an admixture or purified upon harvesting. Emerging research demonstrates the ability of bone marrow-derived stem cells, upon injury, to mobilize from the bone marrow (BM) into the peripheral blood. This feature is very practical for harvesting cells, which is currently employed for many immunologic, hematologic, and oncologic clinical applications. With relation to stroke, once in systemic circulation they may migrate to regions of the central nervous system in response to neuronal injury [35]. Cellular components of bone marrow include: hematopoietic stem cells (HSCs), mesenchymal stem cells (MSCs), endothelial progenitor cells (EPCs), and very small embryonic-like stem cells (VSELs) [36]. Here we will outline the aforementioned cell lines in greater detail.

Hematopoietic stem cells are found primarily in the bone marrow where they give rise to both the myeloid and lymphoid lineages of blood cells. Cytokines produced by the CNS can incite hematopoietic stem cell mobilization into the blood, from the marrow, in response to a cerebrovascular accident (CVA) [37,38,39,40]. This mobilization may also be influenced by neurotransmitters, notably catecholamines, either through a paracrine mechanism signaling directly into the bone marrow or through systemic sympathetic release into circulation [41]. This cytokine-mediated recruitment of HSCs is applied clinically through treatment with granulocyte-colony stimulating factor [39,40].

Abundant mobilization of immature hematopoietic CD34^+^ colony-forming cells and Long-Term Culture-Initiating Cells (LTC-IC) has been observed from clinical data of acute stroke, with the magnitude of this mobilization correlating with recovery of function. Autologous infusions of bone marrow mononuclear cells in human stroke patients during acute, subacute, and chronic phase of stroke have demonstrated no adverse effects of transplantation [42,43,44,45]. Transplantation of HSCs into animal models of stroke has greatly elucidated the therapeutic benefits of this type of stem cell in regenerative medicine. HSCs intravenously administered were able to increase the survival rate of stroke mice, accompanied by decreased neuronal cell death, and facilitated recovery from paralysis and forelimb weakness [46]. In an effort to reveal the mechanism of action of HSC therapeutic benefit in stroke, HSCs were intravenously administered at 24 h after ischemic stroke in mice which showed grafted cell migration into the spleen and later into ischemic brain parenchyma, expressing microglial but no neural marker proteins [47]. Moreover, transplanted stroke animals displayed significantly smaller infarct volumes and less apoptotic neuronal cell death in peri-infarct areas accompanied by a reduction of invading T cells and macrophages and a downregulated proinflammatory cytokine and chemokine receptor gene transcription within the spleen [47]. These findings indicate that transplanted HSCs exert therapeutic effects in stroke possibly acting via regulation of both central and peripheral (*i.e.*, spleen) inflammation. Bone marrow (BM) derived HSCs are also being considered as potential treatment for diseases affecting cardiovascular tissue, bone, and cartilage among other tissues.

Mesenchymal stromal cells were first isolated in bone marrow, but have since been found in nearly every tissue of the body. Here we address the therapeutic application of mesenchymal stromal cells, as well as non-bone marrow derived stem cells, for treatment of stroke.

Transplantation of mesenchymal stromal cells into stroke models induces functional recovery of neurological deficits following cerebral ischemia [48,49,50]. The limited differentiation capacity of mesenchymal stromal cells suggests that observed transplantation benefits may be afforded through activation of endogenous repair pathways by secretion of neurotrophic factors which include brain-derived neurotrophic factor (BDNF) [51], nerve growth factor (NGF) [51], vascular endothelial growth factor (VEGF) [52], basic fibroblast growth factor (bFGF, FGF-2) [52], hepatocyte growth factor (HGF) [48,53], and insulin growth factor-1 (IGF-1) [54]. MSCs may recruit primary stem cells from the subventricular and subgranular zones of the brain to the site of injury, while also dampening apoptosis in the penumbral zone of the lesion [51,52]. A clinical trial of intravenous infusion of autologous BM-derived mesenchymal stromal cells in ischemic stroke patients shows significant functional improvement in infused patients without adverse effects in comparison with non-infused patients [55]. In a long-term 5 year follow up, patients infused with mesenchymal stromal cells demonstrated increased survival rates and greater functional improvement compared to non-infused patients [56]. However, the current transplantation techniques are plagued by very low graft survival rates, and therefore, mode of delivery remains a significant limitation of mesenchymal stromal cell-based therapies for stroke [57]. 

Mesenchymal stem cells have recently been the focus of many research endeavors, due in large part to their accessibility when compared to other stem cells. Mesoderm-derived mesenchymal stromal cells may be extracted from almost any mesenchymal tissue of the body including: bone marrow, placenta, teeth and adipose tissue. This abundance of harvest sites makes MSCs a preferred line for autologous transplantation. However, evidence indicates that harvest location may impart a specific role to mesenchymal stromal cells as a function of various methods of extraction, isolation, and proliferation [58,59,60,61,62]. To this extent, one site of tissue derived mesenchymal stromal cells may be better qualified for a specific therapy than cells derived from another tissue site. 

Despite their limited differentiation capacity and relatively transient life-span after transplantation, evidence shows that mesenchymal stromal cells promote neurogenesis following ischemic injury [52]. As mentioned previously, benefits may stem from secretion of neurotrophic factors such as BDNF and β-NGF, as well as modulation of vasculature from bone marrow, adipose tissue, skeletal muscle, and myocardium [63].

Laboratory findings indicate that neurotrophic factors are involved in the neuroprotective action of stem cells as evidenced by their trophic effects, but additionally their anti-inflammatory and anti-apoptotic effects in animal models of stroke and other neurological disorders [64,65,66]. That BDNF and NGF have been shown as consistently secreted by transplanted stem cells suggest that targeting these two trophic factors’ signaling pathway may further improve the outcome of stem cell therapy. Alternatively, the combination of trophic factor treatment with stem cell transplantation may allow a more robust functional improvement in the clinic. The safety profiles of these trophic factors in the clinic in other disease indications [67,68], and the recognition of their clinical limitations, will guide the clinical trials of this combination therapy for stroke. Of note, BDNF Val(66)Met polymorphism has been implicated in worsened functional outcome in patients with subcortical stroke [69]. Similarly, serum from stroke patients revealed NGF upregulation significantly correlates with clinical and neuroradiological parameters of brain injury [70].

In addition to the many potential benefits of menchymal strem cell-based therapies mentioned above, there are also significant risk factors that must be addressed. As with many types of stem cells, the risk of mesenchymal stem cells developing into tumors must be considered. One study showed that a sarcoma developed in the lungs of mice following transplantation of mesenchymal stem cells [71]. Not only the cells themselves but also their secretions may affect tumors. Interleukin-6 (IL-6) and vascular endothelial growth factor (VEGF) secreted from MSCs increases the migration of breast cancer cell lines [72]. Breast cancer cells stimulate *de novo* secretion of the chemokine CCL5 from mesenchymal stem cells, which then acts in a paracrine fashion on the cancer cells to enhance their motility, invasion, and metastasis [73]. Accordingly, certain types of mesenchymal stem cells may demonstrate greater tendency toward tumorigenicity and promotion of metastasis.

Endothelial progenitor cells (EPCs) represent a small population of cells present in the blood that give rise to mature endothelium that lines blood vessels. While in circulation, these cells can be recruited to produce new blood vessels, a term called vasculogenesis. 

The etiology of stroke is multifaceted. One contributing factor includes the compromise of vascular integrity, leaving a region vulnerable to stroke. With the endothelium regulating the permeability of the blood brain barrier (BBB), the role of endothelial progenitor cells in producing the mature lining of blood vessels is integral in maintaining cerebral homeostasis. Preliminary studies demonstrated that transplanted EPCs were integrated into newly vascularized endothelium of the hind limbs in ischemic animal models [74]. Further research specifies that BM-derived endothelial progenitor cells are likely signaled to sites of new vascularization prior to differentiation [75,76].

A correlational study in human ischemic stroke patients indicates that the level of circulating EPCs relates to improvement on the National Institute of Health Stroke Scale [77]. Animal models of stroke show that intravenous transplantation of EPCs reduces cerebral infarcts in stroke diabetic mice [78]. Moreover, EPCs can incorporate to the BBB microvasculature and delay the stroke onset in an ischemic hemorrhagic stroke model [79]. In addition, intravenous infusion of autologous EPCs after stroke in rabbits produces functional improvement, decreases number of apoptotic cells, increases microvessel density in the ischemic boundary area, and reduces infarct area [80].

The current hypothesis of very small embryonic-like stem cells is that these pluripotent stem cells are deposited early in embryonic development from an epiblast source, where they function as a reserve that can be accessed in response to physiological stress [81,82]. Investigation is underway using VSELs for stroke therapy in the brain, a region rich in VSEL phenotypic cells [83,84]. VSELs are a great candidate in therapy for cerebral vascular incident because of their potential to differentiate into neurons, oligodendrocytes, and microglia to regenerate damaged CNS [35].

However, current restrictions present a challenge in moving forward. Very small embryonic-like stem cells are present in limited quantity, producing a low yield from harvesting. Such an obstacle may be overcome with refining methods of proliferation prior to transplant [35]. An additional challenge is the decreasing population of VSELs present in older age, further contributing to the difficulty of sufficient yield upon harvesting [84]. 

### 3.2. Harvesting Neural Stem Cells for Neural Repair in Stroke

With endogenous stem cells being located in the subgranular zone (SGZ) of the dentate gyrus, the subventricular zone (SVZ), and the subependymal zone (SEZ) of the spinal cord, the therapeutic potential of NSCs for cerebrovascular accidents seems obvious. Chemokine signals such as stromal-derived factor-1 (SDF-1), vascular endothelial growth factor (VEGF), and angiopoietin are released from ischemic tissue, influencing the course of the SVZ NSCs toward a path along blood vessels to reach the infarcted area [85,86,87,88]. Although endogenous stem cells migrate to the lesion following stroke, there appears to be minimal stem cell survival [89,90,91]. This supports the hypothesis that endogenous neural stem cells may not exert their effects solely by replacement of neuronal tissue, but rather by secreting growth factors that influence repair. Immunological responses may also influence the differentiation of endogenous stem cells. In *ex vivo* studies, microglia from ischemic brains prompted the maturation of NSCs into neurons [92].

Although endogenous NSCs are shown to migrate in response to cellular injury, their effects may be augmented by the addition of exogenous neural stem cells. The literature describes transplantation of NSCs inducing further endogenous stem cell production at the site of injury [93,94,95,96]. However, another study suggests that intravenous infusion of neural progenitor cells decreased neurogenesis despite increasing dendritic length and the number of branch points [97]. This may further support the hypothesis of neurotrophic factors secreted from stem cells exerting a primary effect. 

Neural stem cells are proven in terms of their therapeutic potential; however, they present a few significant limitations. The difficulty of obtaining the cells may be the greatest challenge. Under most circumstances, harvesting neural stem cells would require an invasive procedure for autologous use while allogenic grafts would require a fetal source or manipulation from another cell source. A possibility to circumvent this problem would be harvesting the stem cells for therapy during a surgical procedure already intended for the patient [98], such as during a temporal lobe resection in which subventricular matter, a known source of stem cells, could be harvested. As with many other stem cells, there is constant concern about the potential to illicit aberrant cell growth, producing tumors upon transplantation. Whereas the less differentiated the cell, the less likely it will invoke a host reaction; however, the more naive the stem cell, the greater its propensity for uncontrolled proliferation. 

Adult stem cells possess a reduced capacity for proliferation and may be less tumorigenic. However, this presents a problem with producing a sufficient number of cells for transplantation. To traverse these limitations, researchers have developed methods such as: long-term culturing, immortalization, insertion of oncogenes, or even derivation of neural stem cells from other tissues or from pluripotent stem cells.

### 3.3. Recapitulating Cell Developmental Growth in Other Adult Stem Cells

#### 3.3.1. Mimicking Bone Marrow Therapeutic Transplant Potential: Umbilical Cord Blood (UCB)

With their availability, ease of harvesting, and ability for autologous and allogenic use, the therapeutic potential of umbilical cord blood is expansive. The heterogeneous mixture of cells comprising cord blood includes hematopoietic progenitors, lymphocytes, monocytes, embryonic-like stem cells, and mecenchymal stromal cells. Yet, cord blood is considered immunologically immature and exerts its effects through immune modulation and reducing inflammation [99].

Transplantation of umbilical cord blood-derived stem cells in animal models of stroke has produced encouraging results of functional recovery, reducing infarct size, and higher expression of neuroprotective factors, such as BDNF and VEGF [100,101,102,103]. In other studies, human umbilical cord blood has exhibited protective effects in the rat hippocampus *in vitro*, while promoting dendritic growth. Additional emerging research is investigating the capabilities of human umbilical cord blood hematopoietic stem cells for functional recovery of dopaminergic neuron morphology of the substantia nigra, caudate, and putamen in an 1-methyl-4-phenyl-1,2,3,6-tetrahydropyridine (MPTP) Parkinson’s disease mouse model. After intracardioventricular injection, there was an increase in size and density of tyrosine hydroxylase staining cells of the substantia nigra [104].

#### 3.3.2. Shedding the Fat for Stem Cells: Adipose Tissue

Adipose tissue derived stem cells have demonstrated the ability to differentiate into neural, glial, and vascular endothelial cells, and also show higher proliferative activity with greater production of VEGF and hepatocyte growth factor in comparison with bone marrow derived stromal cells [98]. In combination with the accessibility, these features make adipocyte-derived stem cells a desirable source for neurovascular therapy. Transplantation of adipose-derived stem cells in ischemic stroke models demonstrates reduction in damage [98]. Additional studies exhibited reduced infarct size, improved neurological function, reduced level of cerebral inflammation, and chronic degeneration in an intracerebral hemorrhage model, substantiating their therapeutic value [105,106].

Yet, stem cells derived from adipose tissue are also subject to limitations. It was considered that spontaneous mutations occur with extensive passaging that foster tumorigenesis, potentially leading to cancer [107,108]. Follow-up studies suggest adipose-derived stem cells promote pre-existing cancerous cells, but do not initiate tumorigenesis. In a human clinical trial of spinal cord injury patients, none of the eight patients experienced any adverse events within the three-month follow-up [109].

#### 3.3.3. Gender-Specific Stem Cells: Menstrual Blood-Derived Stem Cells

Following many of the factors considered in harvesting stem cells, menstrual blood provides a source with many benefits. With the monthly cycling of the endometrium, the ease and availability for harvesting is a large benefit in the research for therapeutic potential. Stem cells collected from menstrual blood demonstrate multipotency and secrete trophic factors such as VEGF, BDNF, and NT-3 in response to oxygen glucose deprivation (OGD) in an *in vitro* model of stroke. In such studies, the co-culturing of rat primary neurons with menstrual blood, or its conditioned media, improved survival rate [110]. Further, both intracerebral and intravascular transplantation of menstrual blood-derived stem cells in rat stroke models also enhanced survival and behavioral function [110]. Of note, it has been observed that the adherent fraction of menstrual cells do not lose their karyotypic normality or develop tumorigenic potential even after being expanded through 68 doublings [111].

#### 3.3.4. Mother Knows Best: Breast Milk-Derived Stem Cells

Mammary stem cells (MaSCs) present in tissue of the breast, along with differentiated cells, enter the milk through lactating epithelium. Researchers postulate that these cells enter the breast milk through a combination of migration, cell turnover, and mechanical shearing forces [112,113]. The stem cells of breast milk demonstrate pluripotency similar to that of embryonic stem cell morphology and phenotype and thus allow for differentiation into all three germ layers *in vitro* [113]. Future research may elucidate therapeutic potentials in line with those of ESCs. Additional benefits results from the noninvasive harvesting of the cells, availability, and potential for autologous transplant. In terms of tumorigenicity of breast milk-derived stem cells, a study has reported that even nine weeks after subcutaneous injection of breast milk-derived stem cells in immunodeficient mice, these cells did not produce tumors [113]. Along this line, subpopulations of pluripotent adult cells and other multilineage stem cells have failed to form teratomas. Further work characterizing breast milk-derived stem cells for any oncogenic activity under different pathological conditions is needed to determine their tumorigenicity. 

#### 3.3.5. A Wisdom Tooth: Dental Tissue-Derived Stem Cells

Dental tissue-derived stem cells, such as post-natal dental pulp stem cells (DPSCs) [114], stem cells from exfoliated deciduous teeth (SHED) [115], periodontal ligament stem cells (PDLSCs) [116], stem cells from apical papilla (SCAP) [116,117], and dental follicle precursor cells (DFPCs) [118], which exhibit mesenchymal stromal cell-like capabilities, have been identified (for review, see [119]). Furthermore, dental tissue-derived stem cells have demonstrated differentiation into a variety of cell lines including neural tissue, adipocytes, and odontoblasts [120].

The use of dental tissue-derived stem cells have been utilized in the study of animal model middle cerebral artery occlusion (MCAO), demonstrating improved motor function following transplantation into the dorsolateral striatum [121]. 

#### 3.3.6. Reverting Differentiated Tissues to Stem Cells: Induced-Pluripotent Stem Cells

Once considered unidirectional, stem cells were thought to progress through a linear maturation process leaving them terminally differentiated. However, current evidence suggests otherwise. Through manipulation, differentiated stem cells may be coerced into a prior state of multipotency. Utilizing the method of transfecting specific transcription factors fibroblasts can be manipulated into their embryonic-like stem cell precursors [122]. This technique has also been applied to umbilical cord blood cells, placental mesenchymal stromal cells, neural stem cells, and adipose-derived precursor cells to increase their potency [123,124]. Further studies in animal models of ischemic stroke demonstrate that some of the benefits in transplanting induced pluripotent stem cells (iPSCs) includes improving sensorimotor functions [125,126], reducing infarct size, reducing pro-inflammatory cytokines, and increasing anti-inflammatory cytokines [125].

As noted above, there is speculation for concern when transplanting less differentiated cells. Of particular apprehension is their potential for tumorigenesis and immunogenicity. The transfection technique used to induce retrograde manipulation utilizes transcription factors of known oncogenicity. The finding that transplantation of iPSCs into ischemic brain tissue produces a higher incidence of tumors than in healthy brain tissue, further supports this notion [127]. Transplantation is also limited by rejection by the host, even when autologous cells are grafted [128]. 

With the aforementioned concerns in mind, emergent research is demonstrating the therapeutic feasibility of vector-free and transgene-free induced pluripotent cells while reducing their tumor potential. A current study investigates the use of these human iPS cell-derived neural progenitor cells (hiPS-NPCs) in a mouse ischemic stroke model after discovering they differentiated into functional neurons *in vitro*. There was no evidence of tumor formation for 12 months following *in vivo* transplantation [129].

## 4. Stem Cell Therapy is Not a Magic Bullet: Exploring Co-Adjunctive Therapies

Due to distinct therapeutic potential of individual cell lines, the possibility exists to combine their respective benefits in targeting disease. Mounting literature substantiates the potential for synergistic effects on stem cell survival when co-transplanted. One such study established enhanced stem cell survival when delivered with adipose-derived stem cells [130]. Moreover, co-transplantation therapy may also decrease adverse events. The co-transplantation of bone marrow-derived stromal cells with embryonic stem cells reduced the incidence of tumor production and transplanting neural stem cells with epithelial cells enhanced survival while promoting differentiation [131,132].

The ability to enhance therapeutic effects is not limited solely to the use of stem cells. Combination therapy employs the addition of a substrate to enhance the efficacy of the stem cell line being transplanted. Examples include: Combining bone marrow-derived stromal cells with trophic factors to enhance survival and potentiation [133] or providing a scaffold for stem cell adherence [134].

The recognition of immunosuppressant factors secreted by certain cells (such as bone marrow and Sertoli cells) supports the use of co-transplantation with an immune-protective cell to allow better graft survival of the cells [135]. Sertoli cells are the germ cells of the testis and it has been shown that they are able to secrete trophic factors that are highly immunosuppressive, and which serve as neuroprotective factors in different animal models of neurological disorders [135,136,137]. As discussed previously, with unique sets of growth factors secreted by stem cells, co-transplantation of stem cells should generate a cocktail of growth factors to be secreted and delivered to the injured brain, thereby affording much more improved therapeutic outcomes. Furthermore, following brain injury, different cell types die or succumb to neurodegeneration, thus warranting the need to transplant multiple cell types. In this case co-transplantation of cells that could differentiate into these multiple cell types will be a logical approach towards replacement of the variety of cells damaged after brain injury [138]. A recent study has demonstrated that neural progenitor cell (NPC) survival and therapeutic support can be enhanced when co-grafted with other genetically modified doxycycline NPCs that can provide bFGF when activated [139,140,141].

As combinations for therapy continue to surface and demonstrate effectiveness, many variables still persist. Factors including: optimal dose, route of administration, and sex of donor/recipient, all of which are likely to be contingent upon the cell type being investigated. To date, we have investigated many of these parameters with umbilical cord blood for conditions such as Alzheimer’s disease, Amyotrophic Lateral Sclerosis (ALS), and Sanfilippo syndrome [142], however, there is still much to be ascertained in regards to stroke therapy. To this end, the Stem Cell Therapies as an Emerging Paradigm in Stroke (STEPS) was initiated to resolve these issues and standardize procedures [143,144,145,146]. 

## 5. Conclusions

Throughout this review we discussed how each cell line under investigation has its own unique benefits and limitations associated with use. Some of those limitations are immunogenicity, tumorigenicity, ease of harvesting, and the ability to proliferate cells. Researchers are currently addressing these issues through many of the techniques reviewed; yet there are still limited clinical trials. More so, current research is expanding beyond a single cell line transplant. It is likely that future clinical therapy may include the use of co-transplantation and combination therapy mentioned. Further studies also aim to explore the molecular mechanism of response by native tissue in the presence of stem cells. This may progress the exploration of unique trophic factors produced by the stem cells and their utilization in these novel therapies. In moving forward, research must still be conducted in assessing factors for optimal transplantation parameters and the efficacy of treatment, however, across the literature, it is evident that stem cells provide a promising niche of therapeutic potential.
